# Assessment of Baroreflex Sensitivity Using Time-Frequency Analysis during Postural Change and Hypercapnia

**DOI:** 10.1155/2019/4875231

**Published:** 2019-02-03

**Authors:** Agnieszka Kazimierska, Michał M. Placek, Agnieszka Uryga, Paweł Wachel, Małgorzata Burzyńska, Magdalena Kasprowicz

**Affiliations:** ^1^Department of Biomedical Engineering, Faculty of Fundamental Problems of Technology, Wroclaw University of Science and Technology, Wroclaw 50-370, Poland; ^2^Department of Control Systems and Mechatronics, Faculty of Electronics, Wroclaw University of Science and Technology, Wroclaw 50-372, Poland; ^3^Department of Anesthesiology and Intensive Care, Wroclaw Medical University, Wroclaw 50-556, Poland

## Abstract

Baroreflex is a mechanism of short-term neural control responsible for maintaining stable levels of arterial blood pressure (ABP) in an ABP-heart rate negative feedback loop. Its function is assessed by baroreflex sensitivity (BRS)—a parameter which quantifies the relationship between changes in ABP and corresponding changes in heart rate (HR). The effect of postural change as well as the effect of changes in blood O_2_ and CO_2_ have been the focus of multiple previous studies on BRS. However, little is known about the influence of the combination of these two factors on dynamic baroreflex response. Furthermore, classical methods used for BRS assessment are based on the assumption of stationarity that may lead to unreliable results in the case of mostly nonstationary cardiovascular signals. Therefore, we aimed to investigate BRS during repeated transitions between squatting and standing in normal end-tidal CO_2_ (EtCO_2_) conditions (normocapnia) and conditions of progressively increasing EtCO_2_ with a decreasing level of O_2_ (hypercapnia with hypoxia) using joint time and frequency domain (TF) approach to BRS estimation that overcomes the limitation of classical methods. Noninvasive continuous measurements of ABP and EtCO_2_ were conducted in a group of 40 healthy young volunteers. The time course of BRS was estimated from TF representations of pulse interval variability and systolic pressure variability, their coherence, and phase spectra. The relationship between time-variant BRS and indices of ABP and HR was analyzed during postural change in normocapnia and hypercapnia with hypoxia. In normocapnia, observed trends in all measures were in accordance with previous studies, supporting the validity of presented TF method. Similar but slightly attenuated response to postural change was observed in hypercapnia with hypoxia. Our results show the merits of the nonstationary methods as a tool to study the cardiovascular system during short-term hemodynamic changes.

## 1. Introduction

Baroreflex is a homeostatic mechanism responsible for short-term regulation of arterial blood pressure (ABP) [1]. As it constitutes one of the most important elements of neural control over the cardiovascular system, the assessment of baroreflex function is commonly used to evaluate the overall state of the autonomic nervous system (ANS) [2, 3]. The key components of baroreflex are arterial baroreceptors—specialized mechanoreceptors located in the carotid sinuses and the aortic arch which respond to stretching of arterial walls caused by changes in ABP [4]. Baroreflex operates in a form of negative feedback loop. Increases in beat-to-beat ABP lead to sympathetic deactivation and parasympathetic activation, which in turn results in reduction of heart rate (HR), cardiac contractility, vascular resistance, and venous return. Conversely, decreased ABP leads to rises in HR as well as increases in cardiac contractility, vascular resistance, and venous return caused by sympathetic activation [1, 5]. The magnitude of baroreflex-mediated responses depends on diverse factors, including age [6], posture [7–9], and presence of conditions such as hypertension, myocardial infarction, or chronic heart failure [5, 10].

The effect of posture on baroreflex function has been studied fairly extensively over the years, particularly due to the possible relationship between the reflex response from baroreceptors and orthostatic intolerance [11, 12]. It has been hypothesized that diminished baroreflex response contributes to insufficient regulation of ABP changes accompanying the transition from lying down to standing and is at least partially responsible for symptoms experienced by subjects suffering from orthostatic intolerance or its more acute form, orthostatic hypotension [13, 14]. So far the evidence regarding the influence of postural change, either active standing, passive head-up tilt, or lower body negative pressure which simulates the upright position, has been controversial. Some studies showed that, in the supine position, baroreflex sensitivity (BRS) is consistently higher than during orthostasis [7–9, 15–17]; others reported no change in BRS during the maneuver [18–22].

Another frequently studied aspect of baroreflex function is its relationship with other components of ANS control over the cardiovascular and respiratory systems, particularly the chemoreflex, which is responsible for reflex response to changes in arterial O_2_, CO_2_, and pH [23]. It has been shown that the interplay between these two mechanisms is a complex issue, and baroreflex both influences [24–26] and is influenced by chemoreflex activation [27]. The chemoreceptors are commonly divided into peripheral chemoreceptors, which are responsible for most of the chemoreflex response to hypoxia but are also activated by hypercapnia [28], and central chemoreceptors, which are responsible for most of the response to hypercapnia [29]. Accordingly, investigations into the interaction of baroreflex and chemoreflex have been focused on a variety of conditions, including both hypoxic and hyperoxic hypercapnia [27, 30–32].

One of the factors limiting the scope of experiments aimed at evaluating baroreflex function was the available method of measurement. The regulatory function of baroreflex is assessed using a parameter called baroreflex sensitivity (BRS), which quantifies the relationship between changes in HR and changes in ABP that induced them [33]. Since the introduction of BRS to the clinical setting, various techniques have been suggested to estimate this parameter. The most widely used procedures are based on acute changes in ABP caused by administration of vasoactive drugs [34] or, in a noninvasive approach, the Valsalva maneuver [35] or manipulation of carotid baroreceptors with the neck chamber device [36]. These techniques allow for estimation of BRS from the slope of the regression line between changes in HR (usually expressed in terms of R-R interval) and changes in ABP [5]. They rely on producing large alterations in ABP, and as such, approximate open loop conditions where the change in ABP is driven almost entirely by the external stimulus [37]. To remove the need for external influence on ABP, a number of methods focused on spontaneous oscillations in HR and ABP of corresponding frequency was proposed [38–40]. They are based on the assumption that small, naturally occurring variations in ABP induce baroreflex-mediated response in the same way as acute changes, and therefore, they allow for estimation of BRS [5]. Several such techniques have been proposed over the years, and it has been shown that they produce results in satisfactory agreement with classical approaches in a variety of conditions [10].

More recently, the focus has shifted to techniques better suited for the investigation of nonstationary mechanisms. As is the case with many physiological processes, the signals corresponding to the function of the cardiovascular system are inherently nonstationary, and the classical methods of spectral analysis which operate under the assumption of stationarity may be an inadequate approach unless limited to strictly steady-state experiments [39]. One of the methods that overcome this limitation is the joint time and frequency (TF) approach which allows for changes in spontaneous fluctuations in ABP and HR to be tracked in time [41]. As such, it permits the study of baroreflex as a dynamic rather than static mechanism [42], thereby expanding the range of available tests to hemodynamic maneuvers where transient changes in physiological signals cannot be assessed by standard techniques.

One of such tests, the squat-stand maneuver, has been introduced to studies on baroreflex function as an alternative to classical methods of inducing acute changes in ABP [43]. As the transition from squatting to standing is a form of powerful orthostatic stress accompanied by a large decrease in ABP, it has been proposed that the analysis of relative changes in ABP and R-R intervals during the maneuver may allow for the estimation of BRS value in the same way as the slope between ABP and R-R intervals in the pharmacological or neck chamber methods, but with a noninvasive procedure more closely resembling physiological conditions [44, 45]. Consequently, the changes in hemodynamic parameters that occur during squat-to-stand and stand-to-squat transitions have been well characterized [43, 46]. The procedure itself, however, has not been used to study dynamic BRS assessed using a nonstationary approach or in combination with hypercapnia.

In this paper, a method of BRS assessment in TF domain, introduced by Orini et al. [41], was adapted for the purpose of studying baroreflex as a time-varying mechanism in healthy volunteers during squat-stand test. Many previous studies have focused on differences in baroreflex function between body positions [7–9, 15, 16, 18–21] as well as on the effect of chemoreflex activation associated with changes in blood gases on baroreceptor-mediated responses [23–25, 27, 30–32]. Despite evidence showing the interaction between baroreflex and chemoreflex, little attention was given to the combination of these two factors [11, 47]. Additionally, due in part to the relative novelty of dynamic BRS assessment, the dynamic nature of baroreflex function remains poorly characterized. The aim of this work was to investigate the effect of postural changes combined with normal partial pressure of CO_2_ (normocapnia) and increased partial pressure of CO_2_ accompanied by decreased partial pressure of O_2_ (hypercapnia with hypoxia) on baroreflex response and corresponding hemodynamic parameters. Our study offers new insights into the time-variant properties of baroreflex function during strong orthostatic stress caused by transition from squatting to standing under conditions of chemoreflex stimulation.

## 2. Materials and Methods

### 2.1. Study Group

Forty healthy volunteers (20 female, 20 male; median age: 22 years; range: 18–31 years) participated in the study. The following exclusion criteria were applied: tobacco smoking; cardiovascular, respiratory, or neurological diseases; chronic illness or treatment known to influence cardiovascular parameters. Before participation, all subjects underwent basic medical examination to test for possible disorders. The subjects were asked to abstain from alcohol and caffeine for at least 12 hours before the experiment.

### 2.2. Data Acquisition

Beat-to-beat ABP was measured noninvasively using a plethysmographic system (Finometer® MIDI, FMS Medical Systems, Amsterdam, The Netherlands) from a cuff placed on the middle finger of the subject's left hand. During the experiment, the subject's hand was held at heart level. End-tidal carbon dioxide level (EtCO_2_) and respiration rate (RR) were measured using a standard capnograph (RespSense™, NONIN, Plymouth, MA, USA) connected to a breathing mask fitted to the subject's nose and mouth. All signals were recorded with the sampling frequency of 200 Hz using ICM+® data acquisition software (Cambridge Enterprise Ltd, Cambridge, UK).

### 2.3. Experimental Protocol

**Figure 1:**
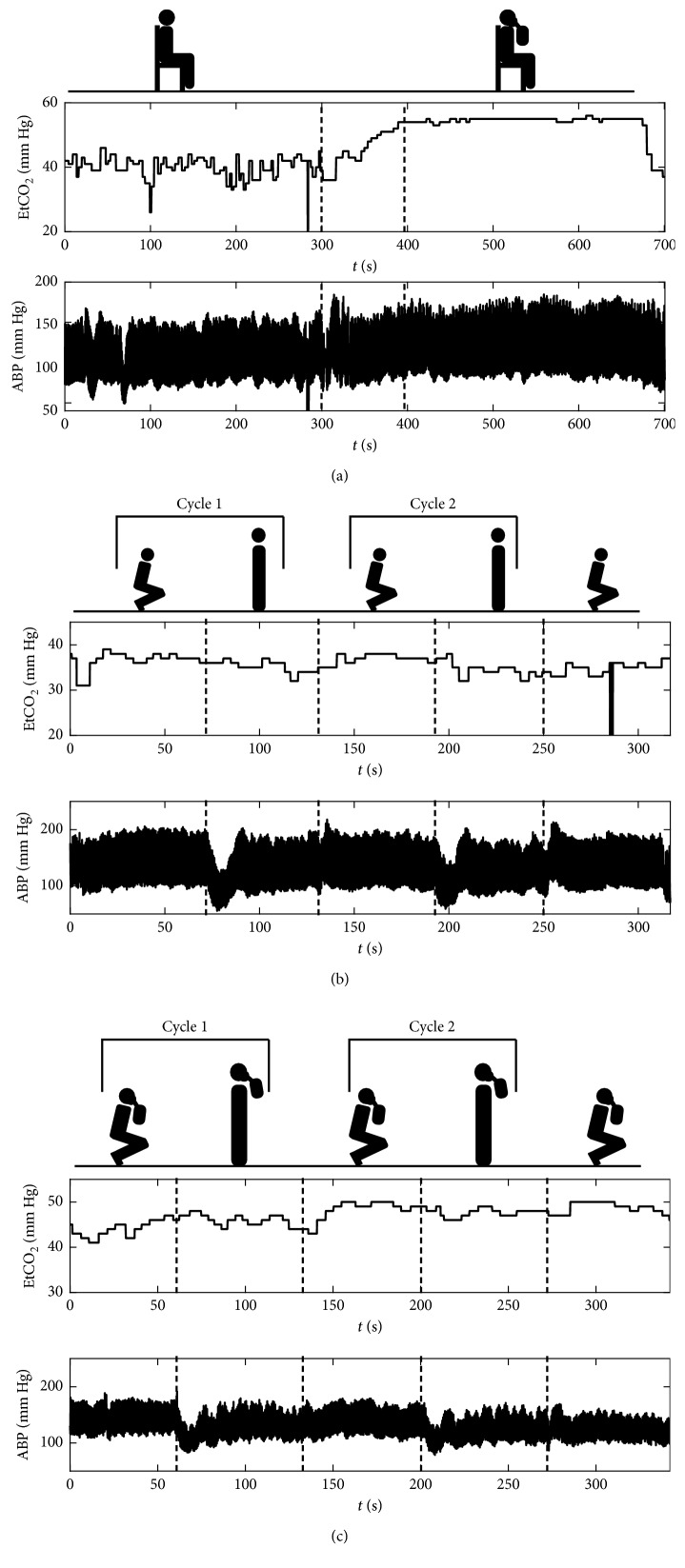
Exemplary recordings of end-tidal CO_2_ level (EtCO_2_; upper plots) and arterial blood pressure (ABP; lower plots) signals from the performed experiment: (a) resting position in normocapnia and hypercapnia with hypoxia, (b) postural change in normocapnia, and (c) postural change in hypercapnia with hypoxia. Figures above the plots indicate the subject's body position and presence of rebreathing bag during each part of the measurement. Dashed vertical lines denote approximate times of change in EtCO_2_ level or body position.

The experiment consisted of three separate measurement stages: (a) normocapnia and hypercapnia with hypoxia in resting position, (b) postural change in normocapnia, and (c) postural change in hypercapnia with hypoxia. After each stage of the experiment, there was a 5-minute rest period to let the measured signals return to their initial values. During the first stage, data were collected in the sitting position. Initially, the subject was asked to breathe spontaneously for about 5 minutes. Then, the subject was asked to rebreathe with a 1.5 L plastic reservoir bag attached to their face mask which resulted in an increase in the EtCO_2_ level up to hypercapnia (EtCO_2_ > 45 mm·Hg). After the subject reached hypercapnic levels of EtCO_2_, another 5 minutes of data were recorded, after which the bag was disconnected and the subject was asked to breathe spontaneously again (Figure 1(a)).

In the postural change stages, the subject was asked to alternate between the squatting and standing position at 60-second intervals marked by a metronome for approximately 5 minutes, starting from squatting, which constituted two full cycles of squat-to-stand and stand-to-squat transitions (Figure 1(b)). Data were collected continuously throughout the whole stage. During normocapnia, the subject was instructed to breathe normally. Hypercapnia was again achieved by rebreathing through a reservoir bag attached to the subject's face mask. In the hypercapnia stage, change in body position started after hypercapnic level of EtCO_2_ was reached. Exemplary recording in hypercapnia is presented in Figure 1(c).

Ethical approval for this study was obtained from the Bioethical Committee of Wroclaw Medical University (permission no. KB–170/2014). The study protocol was in accordance with the tenets of the Declaration of Helsinki. Written informed consent was obtained from all individual participants included in the study.

### 2.4. Signal Analysis

The method for TF assessment of baroreflex function is a modification of an earlier algorithm proposed by Orini et al. [41]. Presented approach is based on Zhao–Atlas–Marks (ZAM) distribution [48] in opposition to smoothed pseudo-Wigner–Ville distribution used in the original method. The ZAM distribution was selected due to its property of relatively high suppression of cross-terms in the TF representations. A ZAM-based framework for estimation of TF representations of power spectra, coherence, and phase shift was presented in detail in our previous work [49] and used in assessment of cerebral autoregulation [49, 50]. Here, the framework was adapted for the purpose of TF BRS estimation. All analyses were performed using programs custom written in MATLAB® (MathWorks®, Natick, MA, USA).

#### 2.4.1. Extraction of Analysis Periods

In the resting position stage, two data segments were manually extracted from each measurement session, one corresponding to normocapnia and the second corresponding to hypercapnia with hypoxia (see Figure 1(a)). These two segments were later analyzed as individual recordings. In the postural change stages, all of collected data (i.e., including both squatting and standing periods) were treated as one recording (see Figures 1(b) and 1(c)). The last squatting period was later excluded from the analysis due to incompleteness of data in some subjects.

#### 2.4.2. PIV and SAPV Time Series

The noninvasive recording of ABP from each measurement was used to obtain estimates of pulse interval variability (PIV) and systolic arterial pressure variability (SAPV) that represent changes in HR and ABP associated with the cardiac baroreflex response arc. Most commonly, the beat-to-beat interval for calculation of BRS is derived from the ECG signal [33]. Due to the nature of presented experiment, mainly the postural changes which pose an obstacle to obtaining reliable, artifact-free ECG recording during the whole measurement from electrodes attached to the subject's skin, the ABP signal was chosen instead. Earlier comparative studies showed that in the event of ECG signal unavailability, distal pulse wave can be used as an acceptable alternative as a marker of cardiac rhythm [51], and the substitution does not significantly influence BRS estimates [33].

First, systolic peaks were identified as the local maxima of ABP signal and characterized by their temporal location, *t*_*n*_, and value, ABP(*t*_*n*_). The pulse interval was calculated as the time interval between two consecutive peaks (PI(*t*_*n*_)=*t*_*n*+1_ − *t*_*n*_). The corresponding systolic pressure was taken from ABP value at time *t*_*n*_. The two values were then assigned to resulting time series (pulse interval and systolic arterial pressure) at time *t*_*n*_. Due to variable heart rate, the resulting time series were not uniformly sampled. In order to ensure uniform sampling of signals for further analysis, spline interpolation with the sampling frequency of 4 Hz was performed. Final estimates of PIV and SAPV were obtained by high-pass filtering of interpolated pulse interval and systolic arterial pressure signals with a cutoff frequency of 0.03 Hz, as proposed in [41].

#### 2.4.3. Time-Frequency Representations

Following the estimation of PIV and SAPV, ZAM distributions of both signals were calculated [52]. The ZAM distribution cross spectrum of signals *x* and *y* in the form employed in this study is described by the following formula [53]:

(1) $$\begin{array}{c} S_{\text{ZAM},xy}\left(t,f\right)=\int_{-\infty }^{\infty }h\left(\tau \right)e^{-j2\pi f\tau }\left[\int_{t-\left|\tau \right|/2}^{t+\left|\tau \right|/2}g\left(u-t\right)x\left(u+\frac{\tau }{2}\right)y^{*}\left(u-\frac{\tau }{2}\right)\;du\right]\;d\tau , \end{array}$$

where *g* and *h* are the so-called window functions responsible for smoothing in time and frequency domain. Correspondingly, the autospectra of signals *x* and *y* are denoted as *S*_ZAM,*xx*_(*t*, *f*) and *S*_ZAM,*yy*_(*t*, *f*).

Size of the windows is a factor that influences the temporal and spectral resolution of calculated TF representations. In order to obtain satisfactory resolution with sufficient reduction of interferences for the purpose of BRS estimation, the length of windows was tuned using a procedure presented in detail in our earlier work [49]. Here, the Hanning window of length equal to 2053 samples was selected for *g* and the Hanning window of length equal to 113 samples was selected for *h*.

Two spectral bands which are believed to represent the characteristics of the ABP control system itself as well as the influence of respiratory activity [39] were subsequently extracted from TF representations: low-frequency range (LF; 0.04–0.15 Hz) and the respiration-related high-frequency range (HF; 0.15–0.4 Hz) [41]. The extraction procedure was repeated for TF coherence and TF phase shift described below, and accordingly, final LF and HF estimates of BRS were calculated separately (denoted LF BRS and HF BRS, respectively). Resolution of smoothed representations was estimated as proposed in [54] based on TF representations of model signals: discrete Dirac delta and complex exponential for the temporal (Δ*t*) and frequency (Δ*f*) resolution, respectively. The temporal resolution was estimated at two frequencies: 0.1 Hz and 0.25 Hz, which are located approximately in the middle of the LF and HF spectral bands. The following results were obtained for selected window length: Δ*t* = 3.25 s for the 0.1 Hz band and Δ*t* = 1.25 s for the 0.25 Hz band, and Δ*f* = 0.08 Hz.

**Figure 2:**
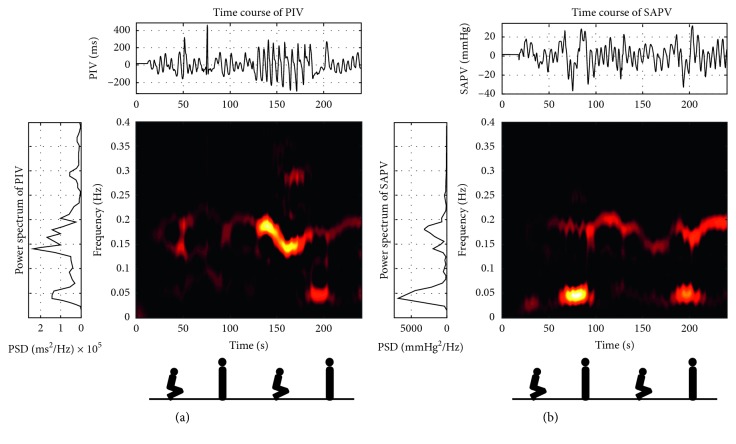
Exemplary time (top plots), frequency (left plots), and time-frequency (centre graphs) representations of (a) pulse interval variability (PIV) and (b) systolic arterial pressure variability (SAPV) signals recorded during postural change in normocapnia stage of the experiment. The low (0.04–0.15 Hz) and high (0.15–0.4 Hz) frequency ranges used in baroreflex sensitivity estimation are shown together. Figures below the graphs indicate the subject's body position during each part of the measurement.

Exemplary TF representations of PIV and SAPV signals from the postural change in normocapnia experiment are shown in Figure 2. Increased energy of TF representations and peaks in power density spectra around the 0.2 Hz frequency range are related to the respiration frequency of the subject.

#### 2.4.4. Time-Frequency Coherence

**Figure 3:**
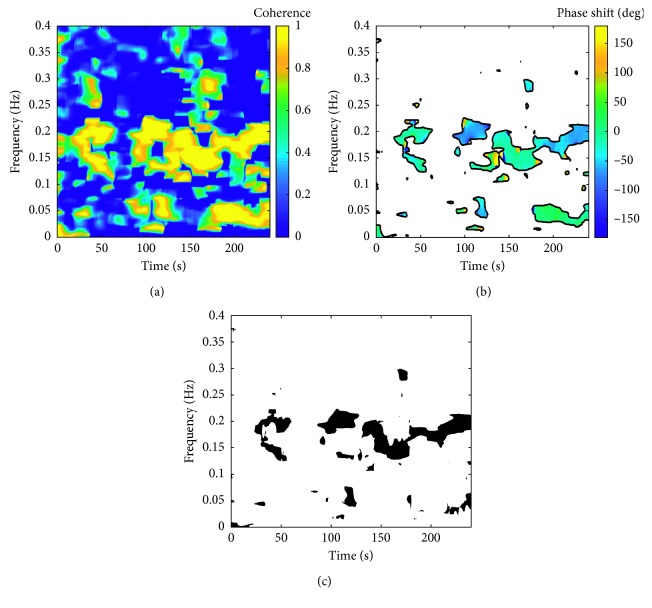
Exemplary representations of TF coherence (TFCoh), TF phase shift (TFPS), and TFCoh-TFPS binary mask for the same subject as in Figure 2 (postural change in normocapnia stage of the experiment). (a) Exemplary representation of TFCoh between pulse interval variability (PIV) and systolic arterial pressure variability (SAPV) signals. (b) Exemplary representation of TFPS between PIV and SAPV signals. White areas denote parts of the representation where TFCoh is lower than 0.9. (c) Binary mask *M* based on TFCoh and TFPS representations. Black areas denote parts of the representation which fulfil the following criteria: (1) TFCoh > 0.9 and (2) TFPS < 0. The low (0.04–0.15 Hz) and high (0.15–0.4 Hz) frequency ranges used in baroreflex sensitivity estimation are shown together.

In general, coherence is a measure of coupling between two signals in the frequency domain. For nonstationary signals, classical spectral coherence may be inadequate due to loss of temporal information. To account for nonstationarity of analyzed signals, magnitude-squared TF coherence (TFCoh) was calculated as defined by the formula [55]:

(2) $$\begin{array}{c} \text{TFCoh}\left(t,f\right)=\frac{{\left|S_{\text{ZAM},xy}^{\left(c\right)}\left(t,f\right)\right|}^{2}}{S_{\text{ZAM},xx}^{\left(c\right)}\left(t,f\right)\cdot S_{ZAM,yy}^{\left(c\right)}\left(t,f\right)}, \end{array}$$

where *S*_ZAM,*xy*_(*t*, *f*) is the ZAM cross spectrum between PIV and SAPV and *S*_ZAM,*xx*_(*t*, *f*), *S*_ZAM,*yy*_(*t*, *f*) are the autospectra of PIV, SAPV as described in the previous section. Indices ^*(c)*^ indicate additional smoothing of ZAM spectra which allow for satisfying the requirement that coherence is bounded to range <0, 1> [55]. Based on a procedure presented in our previous work [49], a threshold of 0.9 for TFCoh was introduced to ensure that coupling between two time series is significant at given (*t*, *f*) point. Exemplary TFCoh representation from the postural change in normocapnia experiment (for the same subject as in Figure 2) is shown in Figure 3(a). High coherence band around the 0.2 Hz frequency range is related to the respiration frequency of the subject.

#### 2.4.5. Time-Frequency Phase Shift

TF phase shift (TFPS) between spectral components of PIV and SAPV was calculated based on the ZAM cross spectrum of PIV and SAPV using the following formula [41]:

(3) $$\begin{array}{c} \text{TFPS}\left(t,f\right)=\text{arg}\left[S_{\text{ZAM},xy}\left(t,f\right)\right], \end{array}$$

where TFPS is bounded to range (−*π, π*>. Previously obtained TFCoh spectrum was used to filter out parts of TFPS representation where coupling between signals is insufficient for estimation of meaningful TFPS values. Only (*t*, *f*) points where TFCoh exceeds threshold value of 0.9 were considered in calculations, as mentioned in the previous section. An in-depth discussion on the methodology of coherence and phase shift estimation used in this study is presented in our previous work [49]. Exemplary TFPS representation from the postural change in normocapnia experiment (for the same subject as in Figure 2) is shown in Figure 3(b).

#### 2.4.6. Time-Frequency Baroreflex Sensitivity

In order to identify TF regions where operation of baroreflex can be assumed, a binary mask *M* based on TFCoh and TFPS spectra was created. The area included in subsequent calculations was defined by the following criteria: (a) TFCoh exceeding 0.9 to ensure coupling between analyzed signals and (b) negative TFPS which corresponds to change in SAPV preceding the change in heart rate, which is the definition of baroreflex-mediated changes. Exemplary mask from the postural change in normocapnia experiment (for the same subject as in Figure 2) is shown in Figure 3(c).

Next, a second binary mask *M*_*B*_ was defined to isolate the dominant spectral components within the LF or HF spectral range. First, binary mask *M* based on TFCoh and TFPS representations was applied to the TF cross spectrum *S*_ZAM,*xy*_(*t*, *f*) of PIV and SAPV signals. Then, time-varying spectral bands were localized based on instantaneous spectral peaks *f*_max_(*t*) within the analyzed frequency range using the following equation:

(4) $$\begin{array}{c} f_{max}\left(t\right)=\underset{f\in B\cap M}{\text{arg max}}\left|S_{\text{ZAM},xy}\left(t,f\right)\right|, \end{array}$$

where *B* is the frequency range (LF or HF) and *M* is the TFCoh-TFPS mask. The mask *M*_*B*_ included (*t*, *f*) points which fulfilled the criterion:

(5) $$\begin{array}{c} f=f_{\text{max}}\left(t\right)\pm \frac{\Delta f}{2}, \end{array}$$

where Δ*f* is the term related to frequency resolution, estimated with a procedure proposed in [54].

**Figure 4:**
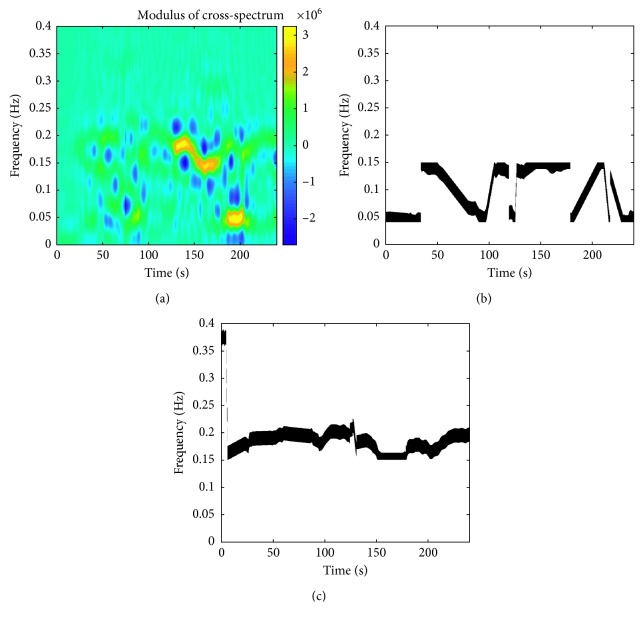
Exemplary representations of modulus of TF cross spectrum and TFCoh-TFPS/band binary masks for the same subject as in Figure 2 (postural change in normocapnia stage of the experiment). (a) Exemplary modulus of TF cross spectrum of pulse interval variability (PIV) and systolic arterial pressure variability (SAPV) signals. (b) Binary mask *M*_LF_ based on TFCoh-TFPS mask and instantaneous spectral peaks of TF cross spectrum in low-frequency band (0.04–0.15 Hz). (c) Binary mask *M*_HF_ based on TFCoh-TFPS mask and instantaneous spectral peaks of TF cross spectrum in high-frequency band (0.15–0.4 Hz). In (b) and (c), black areas denote parts of the representations used in BRS estimation.

The binary mask *M*_*B*_ was then applied separately to TF representations of PIV and SAPV. Final time course of BRS values was estimated using the following formula [41]:

(6) $$\begin{array}{c} \text{BRS}\left(t\right)=\sqrt{\frac{\int_{M_{B}}S_{\text{ZAM},xx}\left(t,f\right)\;df}{\int_{M_{B}}S_{\text{ZAM},yy}\left(t,f\right)\;df}}, \end{array}$$

where *S*_ZAM,*xx*_(*t*, *f*), *S*_ZAM,*yy*_(*t*, *f*) are the autospectra of PIV and SAPV, respectively, and *M*_*B*_ is the area extracted from TF representations by combined TFCoh-TFPS/band mask. Exemplary cross spectrum of PIV and SAPV signals and final binary masks *M*_*B*_ for LF and HF frequency bands (for the same subject as in Figure 2) are shown in Figure 4. As could be also seen in Figures 2 and 3, the dominant frequency in the HF range, and correspondingly, the band isolated from the cross spectrum, is the respiration frequency (approximately 0.2 Hz).

#### 2.4.7. Sequence Method of Baroreflex Sensitivity Assessment

In order to validate the results of TF BRS assessment with an approach that does not require stationary data, the sequence method proposed by Parati et al. [38] was used as a second source of BRS estimates for the resting position in normocapnia and hypercapnia with hypoxia experiment. PIV and SAPV time series were calculated using the procedure described in an earlier section. Then, sequences of three or more heartbeats where PIV and SAPV change in the same direction with a one-beat delay (i.e., change in SAPV precedes the change in PIV) and the change exceeds threshold values of 5 ms and 1 mmHg, respectively, were identified in each series. For each SAPV-PIV sequence pair with Pearson correlation coefficient exceeding 0.85, linear regression was performed and the slope of the regression line taken as a BRS estimate. All values obtained from a recording were then averaged to produce a single BRS estimate.

#### 2.4.8. Physiological Parameters

In addition to baroreflex sensitivity measures, time courses of hemodynamic parameters characterizing the operation of baroreflex response arc were calculated. Mean ABP (MAP) was calculated as the average pressure over one cardiac cycle. Pulse pressure (PP) was calculated as the difference between the systolic and diastolic pressure in each cycle. Heart rate (HR) was estimated as the fundamental frequency of ABP signal in 0.5–2.5 Hz range based on the local maximum of ZAM autospectrum of the signal at each time instant *t* and then converted to beats per minute (bpm). Additionally, mean EtCO_2_ and mean RR time courses were calculated using moving average with a 5-second-long window.

### 2.5. Statistical Analysis

#### 2.5.1. Resting Position Experiment

Comparison of measures of baroreflex response and physiological parameters between normocapnia and hypercapnia with hypoxia was based on the median value from whole time course in each stage. Normality of data distributions was tested using Shapiro–Wilk test with a significance level of 0.05. Since for most of tested variables the normality hypothesis was rejected, Wilcoxon signed-rank test was used for analysis of differences between normocapnia and hypercapnia with hypoxia. Additionally, BRS estimates obtained using the TF method were compared with the values obtained using the sequence method by means of correlation analysis with Spearman's rank correlation coefficient and the Bland–Altman method of assessing agreement between measurement techniques.

#### 2.5.2. Postural Change Experiment

For the purpose of statistical analysis, steady state of each squatting and standing period was extracted from the whole recording and analyzed separately. The steady state was defined as the last 20 seconds before the next position shift to exclude the return phase after postural change from comparison [46]. As in the resting position experiment, the median value from time course of estimated parameters was taken for statistical testing. Shapiro–Wilk and Wilcoxon signed-rank tests were used, as described in the previous section. Differences were assessed between squatting and standing positions (separately in normocapnia and hypercapnia with hypoxia) as well as between the same position in two cycles of postural change in order to assess the possible effect of time elapsed from the start of the recording and previous postural changes on the analyzed parameters.

## 3. Results

### 3.1. Resting Position in Normocapnia and Hypercapnia with Hypoxia

The results of statistical testing between normocapnia and hypercapnia with hypoxia stages of the resting position experiment are presented in Table 1. In hypercapnia with hypoxia, statistically significant increases in EtCO_2_, MAP, and PP were observed, as well as very small, albeit statistically significant increase in HR, and a decrease in RR. No statistically significant differences were found in HF BRS despite a marked decrease in the estimator's variance between normocapnia and hypercapnia with hypoxia. However, a statistically significant decrease in LF BRS was observed.

### 3.2. Comparison with the Sequence Method

**Figure 5:**
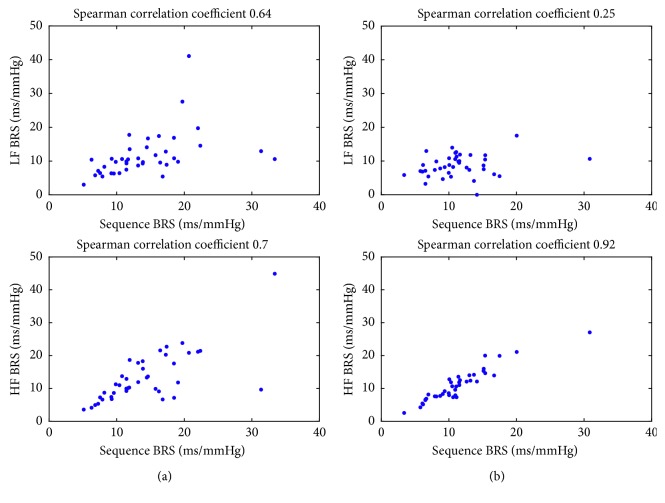
Correlation plots for BRS estimates obtained using the sequence and TF methods for data from the resting position in (a)normocapnia and (b) hypercapnia with hypoxia experiments.

Figure 5 shows correlation plots between BRS estimates obtained using the sequence and TF methods in normocapnia and hypercapnia with hypoxia in the resting position. TF BRS values exhibited a varying degree of correlation with the sequence method depending on analyzed spectral band. LF BRS estimates were moderately to weakly correlated with results from the sequence method (Spearman's correlation coefficient equal 0.64 and 0.25 for normocapnia and hypercapnia with hypoxia, respectively), whereas in the HF band, the correlation coefficient increased to 0.7 and 0.92, respectively. While the degree of correlation remained similar in normocapnia, there were pronounced differences between the measures in hypercapnia.

**Figure 6:**
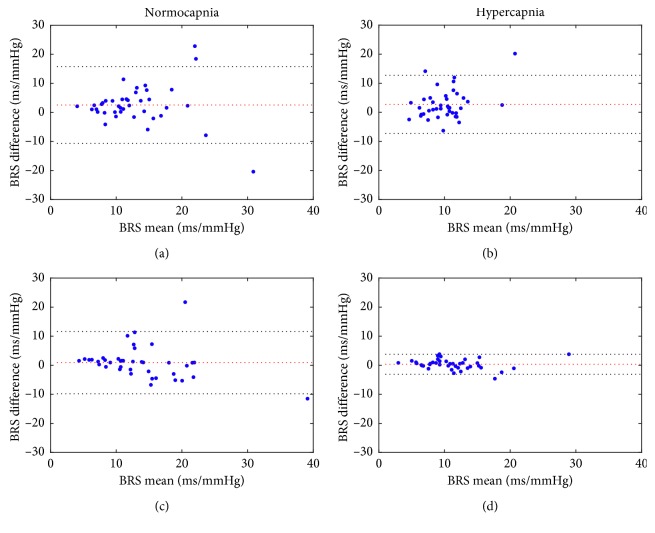
Bland–Altman plots for BRS estimates obtained using the sequence and TF methods for data from the resting position experiment: (a) sequence vs. LF BRS in normocapnia, (b) sequence vs. LF BRS in hypercapnia with hypoxia, (c) sequence vs. HF BRS in normocapnia, and (d) sequence vs. HF BRS in hypercapnia with hypoxia. Dotted red lines indicate mean values of difference between BRS estimates obtained with the methods. Dotted black lines indicate ±1.96 ∗ SD values of difference between BRS estimates obtained with two methods.

The Bland–Altman plots for the TF and sequence BRS estimates are presented in Figure 6. As was the case with correlation, the degree of agreement was highest between the sequence and HF BRS in hypercapnia and similar for LF and HF BRS in normocapnia. There was small but visible positive bias for LF BRS estimates which diminished for HF BRS. Overall, however, the ±1.96 ∗ SD lines for three out of four comparisons (i.e., excluding HF BRS in hypercapnia) indicate at best moderate agreement between methods.

### 3.3. Time Courses of Physiological Parameters and Baroreflex Sensitivity Estimates

**Figure 7:**
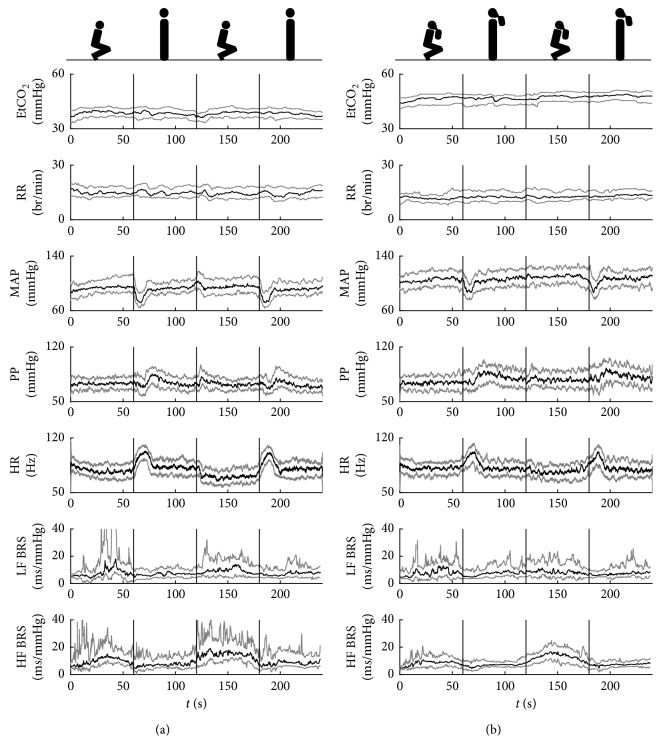
Group-averaged time courses of respiratory and hemodynamic parameters and BRS estimates in (a) normocapnia and (b) hypercapnia with hypoxia. EtCO_2_: mean end-tidal carbon dioxide level; RR: mean respiration rate; MAP: mean arterial blood pressure; PP: pulse pressure; HR: heart rate; LF BRS: baroreflex sensitivity in low-frequency range; HF BRS: baroreflex sensitivity in high-frequency range. Black lines: median; grey lines: first/third quartile. Figures above the plots indicate the subject's body position and presence of rebreathing bag during each part of the measurement. Vertical lines denote approximate times of squat-stand transitions.

Figure 7(a) shows group-averaged time courses of EtCO_2_, RR, MAP, PP, HR, and BRS estimates in both frequency ranges during two cycles of postural change in normocapnia. EtCO_2_ remained approximately constant (c. 40 mm Hg) throughout the whole experiment, and no noticeable fluctuations were observed in RR. Both MAP and HR exhibited large fluctuations related to postural change, with a sharp decrease in ABP and a sharp increase in HR at standing up (at approximately 60 s and 180 s) followed by a stabilization period and then smaller increase in both parameters at squatting (at approximately 120 s; the last change is not presented in the plot). A pattern corresponding to changes in MAP was also found in PP. Similarly, there were pronounced differences between BRS (both LF and HF) levels in the two positions, with higher BRS observed during squatting and lower BRS during standing.

In hypercapnia with hypoxia (Figure 7(b)), no fluctuations were observed in either EtCO_2_ or RR estimates at the time of postural change, but a small, steady progressive increase in EtCO_2_ occurred over the course of the whole experiment. MAP and HR exhibited similar trends as those observed in normocapnia; however, the fluctuations were slightly attenuated, and the peaks at transition from standing to squatting were no longer visible. Conversely, the sharp drops in PP that occurred in normocapnia were diminished, and the overshoot that followed them became more pronounced. The trends in BRS estimates remained similar to normocapnia, but the variance of values (represented here by the first and third quartile lines) visibly decreased.

### 3.4. Postural Change in Normocapnia

The values of physiological parameters and BRS estimates during postural change between squatting and standing in normocapnia are presented in Table 2. In normocapnia, no differences were found for RR, PP, or LF BRS during the second cycle of the experiment, while statistically significant changes between body positions were observed in all other parameters. During both cycles of postural change, MAP decreased, whereas HR increased. A statistically significant decrease was observed in LF BRS during the first cycle of the experiment and in HF BRS during both cycles. Small but significant decreases were also observed in EtCO_2_ during both cycles; however, the absolute values remained within the normocapnia range.

During the second cycle, HF BRS was significantly higher than at the beginning of the experiment (*p*=0.002 for squatting, *p*=0.04 for standing), while HR, MAP, and PP were lower (HR: *p* < 10^−6^ for squatting, *p*=0.04 for standing; MAP: not statistically significant for squatting, *p*=0.02 for standing; PP: *p* = 0.02 for squatting, *p*=0.04 for standing). The other parameters showed no differences with measurement progression.

### 3.5. Postural Change in Hypercapnia with Hypoxia

In hypercapnia with hypoxia (Table 3) during the first cycle of postural change, only PP and HF BRS changed significantly, while during the second cycle, differences were also observed for MAP and HR. No changes in either cycle of the experiment were observed for EtCO_2_, RR, and LF BRS. A significant increase in HF BRS (*p* < 10^−4^), MAP (*p* < 10^−3^), and PP (*p* < 10^−3^)) was observed for the second squatting position compared to the first one. Similarly, EtCO_2_ increased significantly between the same position in the first and second cycle (*p*=0.002 for squatting; *p* < 10^−4^ for standing), which corresponds to the previously mentioned progressive rise throughout the experiment.

**Figure 8:**
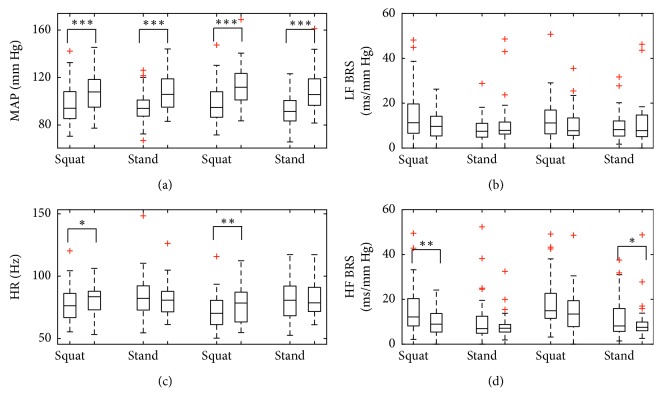
Comparison of hemodynamic parameters and BRS estimates in corresponding stages of postural change in normocapnia and hypercapnia with hypoxia. MAP: mean arterial blood pressure; HR: heart rate; LF BRS: baroreflex sensitivity in low-frequency range; HF BRS: baroreflex sensitivity in high-frequency range. Boxes indicate median (central line) and first/third quartile (edges), and whiskers extend to the most extreme data points excluding outliers marked by + sign. Forks indicate statistically significant differences by Wilcoxon-signed rank test: ^*∗*^*p* <0.05; ^*∗∗*^*p*<0.005, ^*∗∗∗*^*p*<0.0005. In each position, the left-hand side box represents normocapnia and the right-hand side box represents hypercapnia with hypoxia.

Comparison between cardiovascular parameters and BRS estimates in corresponding stages of the experiment in normocapnia and hypercapnia with hypoxia is presented in Figure 8. Statistical analysis supported observations from time courses of individual parameters, with statistically significant differences observed for MAP in all four stages of the measurement, HR in the squatting position and HF BRS in two stages (first squatting position and second standing position; a decrease in HF BRS can, however, be observed in all four stages, as can be a decrease in LF BRS in the squatting position).

## 4. Discussion

The use of the TF approach to BRS assessment permitted us to study baroreflex as a dynamic mechanism during changes in ABP induced by squat-stand maneuver in combination with chemoreflex stimulation by changes in EtCO_2_. Our results are in accordance with previously reported differences in BRS estimates between body positions [7–9, 15–17]. This supports the validity of the TF method based on ZAM distribution as a technique to study the characteristics of baroreflex-mediated response. However, we found moderate correlation between BRS estimated using the proposed method when compared to the well-established sequence method in steady-state normocapnia and hypercapnia with hypoxia. The correlation was higher for HF BRS estimates, which indicates similar relationship to one reported during previous comparative studies under different experimental conditions [33]. The degree of agreement varied between methods and subjects; this observation is however supported by earlier studies which concluded that BRS estimates are not interchangeable between methods of assessments, as each method has its inherent limitations [5, 33].

Furthermore, the inherent ability of the TF approach to follow changes in BRS estimates in time allowed us to relate the time courses of BRS with corresponding time courses of hemodynamic and respiratory parameters during postural change in normal and increased EtCO_2_ conditions. We observed that in hypercapnia with hypoxia, the pattern of hemodynamic response to squat-stand transitions is attenuated but mostly preserved.

### 4.1. Effect of Postural Change on BRS

A number of previous studies have focused on assessing baroreflex function during postural change. Some studies reported that, in the supine position, both classical [7, 8] and spontaneous [9, 15–17] BRS estimates are higher than those in the upright position. Conversely, others showed similar BRS during standing and lying down [18–22]. Detailed investigations separating the cardiovagal component of baroreflex, operating through changes in HR, from the sympathetic arm and its effect on systemic vasculature [14, 20, 21, 56] demonstrated that maintenance of ABP during orthostasis is primarily due to vascular responses, not alterations of HR. The lack of agreement between different studies was partially attributed to inadequacy of the spontaneous methods of BRS assessment in conditions of orthostatic stress.

In an attempt to explain the discrepancies, Schwartz et al. [37] related sigmoidal baroreflex stimulus-response curves obtained using the invasive pharmacological method to BRS estimates based on spontaneous oscillations in ABP and R-R interval. Unlike the methods based on acute changes in ABP, which assess BRS over a range of pressure, the spontaneous methods estimate BRS at a specific operating point of that range [57]. Schwartz et al. established that, in the upright position, baroreflex operates at higher ABP and higher HR, and consequently, the operating point of the reflex moves from the linear central part of the stimulus-response curve to the nonlinear part [37]. Diminished BRS observed in orthostasis is therefore not the effect of a decrease in maximum gain itself but of baroreflex resetting to higher ABP, an effect which has been shown previously in both orthostasis [22] and exercise [57]. Their findings supported the claim that spontaneous BRS is in fact an accurate measure of BRS, in good agreement with the pharmacological method at specific operating points.

Our results are in accordance with Schwartz et al. [37] and earlier studies [7–9, 15, 16] as we observed a significant decrease in TF BRS estimates in the standing position in both cycles of the experiment. TF BRS values averaged over the stabilization period between postural changes correspond to steady-state estimates obtained using standard methods of spontaneous BRS assessment. It is therefore reasonable to assume that the difference in BRS estimates we observed arises from the same resetting mechanism and reflects changes in baroreflex operating point gain. The agreement with previously reported results shows that the TF method based on ZAM distribution correctly identifies the trends in BRS during postural change. The lack of stationarity in the cardiovascular system has been raised in numerous studies over the past decades; accordingly, the need for methods of BRS assessment which account for the nonstationary nature of cardiovascular signals led to formulation of several such approaches [41]. The framework based on ZAM distribution presented in this paper combines the intrinsic ability of TF analysis to identify transient changes in cardiovascular parameters [42] with the advantages of ZAM representation with regard to cross-term suppression and lack of trade-off between time and frequency resolution characteristic to short-time Fourier transform [48]. Moreover, the previously validated [41] statistical approach to localization of regions of interest in TF representations allows for BRS estimation in areas where observed changes can be credibly associated with baroreflex response.

### 4.2. Hemodynamic Response to Squat-to-Stand Maneuvers in Normocapnia

A novel finding is the evolution of TF BRS estimates over time during repeated shifts between body positions. So far, the squat-to-stand maneuver has not been used to study dynamic BRS using TF analysis; earlier investigations focused on the time courses of hemodynamic parameters such as ABP and HR that accompany squat-to-stand and stand-to-squat transitions [58–60]. In our study, we observed a pattern of ABP and HR fluctuations which is in agreement with previous findings [43]. Namely, the transition from squatting to standing results in a marked fall in MAP related to decrease of total vascular resistance and translocation of blood to the lower extremities, which is countered by subsequent increase in HR indicative of baroreflex response. The latter in turn results in ABP recovery to the pretransition level. Conversely, transition from standing to squatting causes a sharp peak in both MAP and HR, followed by sustained increase in ABP and corresponding decrease in HR. The pattern of distinct decrease in ABP and baroreflex response in the form of HR increase after squat-to-stand shift is largely preserved in group-averaged time courses from all subjects (Figure 7). The fluctuations in BRS estimates in both the LF and HF range follow the steady-state stages of the experiment, with maxima in the squatting and minima in the standing position occurring approximately in the final 20 to 30 seconds of each 1-minute interval, and therefore after the recovery period [46]. This does not contradict the association made between the change in spontaneous BRS and baroreflex resetting on the stimulus-response curve. On the contrary, it also shows that TF BRS allows for differentiation of physiological conditions which influence baroreflex function not only based on steady-state averages, but also time courses of the index.

### 4.3. Hemodynamic Response to Squat-to-Stand Maneuvers in Hypercapnia with Hypoxia

In hypercapnia with hypoxia, we observed differences in the time course of hemodynamic parameters and BRS estimates during squat-to-stand transitions, but the general trends appeared to be preserved to a considerable extent when compared to normocapnia. The distinctive pattern of sharp decrease in ABP following the change from squatting to standing and corresponding increase in HR was attenuated, but visible. However, the smaller peak occurring in both signals at stand-to-squat transition was not present, and the magnitude of changes between steady-state periods was considerably smaller for both hemodynamic parameters and BRS estimates. Taken together, these findings would suggest that hypercapnia inhibits the hemodynamic response to postural change. In steady-state experiments, the influence of arterial CO_2_ on baroreflex gain, or more accurately lack thereof, has been relatively well documented. It has been shown in multiple studies that neither mild nor severe hypercapnia has any meaningful effect on BRS estimates with regard to control of heart rate [23, 27, 30, 32, 61], and baroreflex gain is maintained in both hypocapnic and hypercapnic conditions as yet another example of baroreflex resetting [23, 30, 31]. Contrasting results have been presented with regard to hypoxia which accompanied the progressive hypercapnia in our study, with some studies reporting similar baroreflex resetting with no alterations in baroreflex gain [25, 27, 30], but others citing decreases in the parameter [23, 62]. Still, the mechanism underlying the preserved function of baroreflex in hypercapnia and hypoxia has not been fully explained, largely due to the complex interaction between baroreflex and chemoreflex.

There is evidence supporting bilateral interaction between peripheral chemoreceptors and baroreceptors, with inhibition of sympathetic response to hypoxia by baroreflex activation [24–26] on the one hand, and on the other hand, attenuation of baroreflex response by peripheral chemoreceptors [27]. While baroreflex has been shown to have no inhibitory effect on sympathetic activity during central chemoreceptor stimulation [24], baroreflex resetting [30, 31] and changes in baroreflex control of systemic vasculature [23, 27] would suggest the influence of central chemoreflex on baroreflex. If, as proposed earlier, diminished BRS in the upright position in normocapnia is the result of baroreflex resetting, our results obtained in hypercapnia with hypoxia suggest that the resetting mechanism is affected but not completely abolished by simultaneous chemoreceptor activation. It should be noted, however, that we observed differences in the LF and HF baroreflex sensitivity, with more pronounced response to postural change in the HF range. This corresponds to differences in BRS estimates that were found in steady-state normocapnia and hypercapnia with hypoxia and suggests asymmetry in the spectral characteristics of baroreflex that warrants further study.

Moreover, previous studies showed that hypercapnia attenuates sympathetic response to head-up tilt [47] and improves tolerance to orthostatic stress simulated by lower body negative pressure [63]. In our study, we observed in hypercapnia with hypoxia that changes in both time courses and steady-state values of hemodynamic parameters are smaller than their normocapnia counterparts, in agreement with earlier results. It should be noted, however, that the previous studies were primarily conducted as steady-state experiments, comparing supine to upright levels of BRS and hemodynamic parameters, while in our study the full dynamic response was recorded.

### 4.4. Limitations

Firstly, we performed noninvasive measurements of ABP from the subject's finger and used them to derive measures of both systolic pressure and pulse interval variability. The noninvasive recordings have been shown to produce reliable estimates of ABP during standard clinical tests [64], including orthostatic stress [65], and BRS estimation based on noninvasive measurements is viewed as a feasible tool in clinical practice [66, 67]. Moreover, pulse interval is regarded as an acceptable substitute for R-R interval in assessment of heart rhythm [51]. However, the distance between the location of the finger cuff and electrodes commonly used to obtain the ECG signal could introduce a time delay to HR responses which was underestimated in our experimental setting. Secondly, we measured but did not control respiration frequency, and we did not monitor tidal volume; as a result, we cannot quantitatively assess the ventilatory response based on recorded signals. We can only infer that the chemoreceptors were activated by elevated CO_2_ and decreased O_2_. Thirdly, due to the strenuous nature of the procedure and the overall measurement length, the recording time in each stage had to be limited as to reduce the subject's fatigue and allow them to complete the session. For that reason, we only recorded one minute in each body position. Based on previous findings, the latter should, however, be enough to observe the change and recovery periods as well as steady-state periods of the transitions, since most changes in hemodynamic parameters occur in the first 30 seconds after postural change [46].

The TF method employed in this study was primarily selected due to its ability to produce time course of BRS estimates during transient changes in hemodynamic parameters associated with squat-stand transitions. As it is based on spontaneous oscillations in ABP and heart rhythm, albeit assessed with consideration for the nonstationary nature of the cardiovascular system, it is subject to the same limitations as other methods of spontaneous BRS estimation. The indices we obtained are a measure of baroreflex gain at operating point and do not offer information about the full stimulus-response curve [57]. While it has been shown that in orthostatic stress conditions, spontaneous BRS correlates with the pharmacological methods and reveals the effect of baroreflex resetting [37], similar comparison has not been made in hypercapnia. Therefore, any inference we made about the possible influence of the resetting mechanism on trends observed in high EtCO_2_ conditions is rooted in previous observations about postural change or hypercapnia and requires a follow-up study with assessment of the entire response curve. Similarly, estimation of additional cardiovascular parameters, particularly vascular resistance, would be necessary to better characterize the response to squat-stand maneuver in hypercapnia.

Lastly, while the ability to produce time course of BRS estimates is a definite advantage of the TF approach, it is also the reason for its computational complexity. The presented method of TF analysis is not adjusted to long recordings lasting several hours. It is, however, particularly suited to studying baroreflex during short-time hemodynamic tests where standard methods result in loss of information on transient changes, and it offers a combination of nonstationary processing methods with statistical approach to coherence and phase-shift estimation.

## 5. Conclusions

We presented dynamic response of hemodynamic parameters and baroreflex sensitivity estimates assessed using time-frequency analysis to orthostatic stress induced by squat-to-stand maneuvers in conditions of normal and high end-tidal CO_2_. The results show that hypercapnia is associated with differences in cardiovascular response to rapid transitions between body positions which could indicate alterations in hemodynamic adaptation and autonomic control arising from chemoreflex activation. The results warrant further study into cardiac and circulatory modulation during postural changes combined with chemoreceptor stimulation.

## Figures and Tables

**Table 1:** Physiological parameters and baroreflex sensitivity estimates during normocapnia and hypercapnia with hypoxia in resting position.

Parameter	Normocapnia	Hypercapnia with hypoxia	*p* Value
EtCO_2_ (mm Hg)	38 (34–40)	48 (45–50)	<10^−7^
RR (breaths/min)	14 (12–17)	12 (11–14)	<10^−3^
MAP (mm Hg)	88 (78–96)	98 (86–107)	<10^−6^
PP (mm Hg)	67 (57–72)	71 (59–82)	0.002
HR (bpm)	73 (64–78)	74 (68–83)	<10^−3^
LF BRS (ms/mm Hg)	9.2 (7.0–11.0)	8.1 (6.2–9.8)	0.02
HF BRS (ms/mm Hg)	10.7 (8.0–17.7)	10.6 (7.9–13.1)	ns

Values are presented as median (first quartile–third quartile). EtCO_2_: mean end-tidal carbon dioxide level; RR: mean respiration rate; MAP: mean arterial blood pressure; PP: pulse pressure; HR: heart rate; LF BRS: baroreflex sensitivity in low-frequency range; HF BRS: baroreflex sensitivity in high-frequency range; ns: difference not statistically significant.

**Table 2:** Physiological parameters and baroreflex sensitivity estimates during postural change between squatting and standing in normocapnia.

Parameter	Cycle 1			Cycle 2		
	Squatting	Standing	*p* Value	Squatting	Standing	*p* Value
EtCO_2_ (mm Hg)	39 (37–42)	38 (36–42)	0.005	39 (36–41)	38 (36–40)	0.002
RR (breaths/min)	15 (12–18)	14 (12–18)	ns	15 (12–18)	15 (11–17)	ns
MAP (mm Hg)	94 (86–108)	94 (88–101)	0.004	95 (87–108)	91 (84–101)	<10^−4^
PP (mm Hg)	74 (66–82)	73 (66–82)	ns	71 (67–76)	69 (65–80)	ns
HR (bmp)	76 (67–86)	82 (73–92)	0.003	70 (61–81)	81 (68–92)	<10^−3^
LF BRS (ms/mm Hg)	11.3 (6.6–19.7)	7.5 (4.9–11.0)	0.03	11.2 (6.3–17.0)	8.2 (5.4–12.1)	ns
HF BRS (ms/mm Hg)	12.1 (8.1–20.4)	7.0 (4.9–12.5)	0.001	14.9 (11.5–22.7)	8.2 (5.7–16.0)	0.002

Values are presented as median (first quartile–third quartile). EtCO_2_: mean end-tidal carbon dioxide level; RR: mean respiration rate; MAP: mean arterial blood pressure; PP: pulse pressure; HR: heart rate; LF BRS: baroreflex sensitivity in low-frequency range; HF BRS: baroreflex sensitivity in high-frequency range; ns: difference not statistically significant.

**Table 3:** Physiological parameters and baroreflex sensitivity estimates during postural change between squatting and standing in hypercapnia with hypoxia.

Parameter	Cycle 1			Cycle 2		
	Squatting	Standing	*p* Value	Squatting	Standing	*p* Value
EtCO_2_ (mm Hg)	47 (43–49)	47 (43–48)	ns	48 (45–51)	48 (45–51)	ns
RR (breaths/min)	11 (9–14)	13 (10–15)	ns	12 (11–16)	14 (11–17)	ns
MAP (mm Hg)	108 (95–118)	106 (95–119)	ns	112 (101–123)	106 (97–119)	0.008
PP (mm Hg)	75 (66–83)	82 (72–93)	<10^−4^	77 (68–89)	81 (73–94)	0.006
HR (bmp)	83 (73–88)	81 (71–88)	ns	78 (63–87)	79 (72–91)	0.02
LF BRS (ms/mm Hg)	9.7 (5.4–14.2)	7.9 (6.2–11.6)	ns	7.7 (5.6–13.4)	7.8 (5.1–14.7)	ns
HF BRS (ms/mm Hg)	8.9 (5.4–13.7)	7.1 (5.4–8.8)	0.006	13.4 (7.9–19.4)	7.5 (6.0–9.9)	0.002

Values are presented as median (first quartile–third quartile). EtCO_2_: mean end-tidal carbon dioxide level, RR: mean respiration rate, MAP: mean arterial blood pressure, PP: pulse pressure, HR: heart rate, LF BRS: baroreflex sensitivity in low-frequency range, HF BRS: baroreflex sensitivity in high-frequency range, and ns: difference not statistically significant.

## Data Availability

The data used to support the findings of this study are available from the corresponding author upon request.
